# MRI Radiomics Signature as a Potential Biomarker for Predicting *KRAS* Status in Locally Advanced Rectal Cancer Patients

**DOI:** 10.3389/fonc.2021.614052

**Published:** 2021-05-07

**Authors:** ZhiYuan Zhang, LiJun Shen, Yan Wang, Jiazhou Wang, Hui Zhang, Fan Xia, JueFeng Wan, Zhen Zhang

**Affiliations:** ^1^ Department of Radiation Oncology, Fudan University Shanghai Cancer Center, Shanghai, China; ^2^ Department of Oncology, Shanghai Medical College, Fudan University, Shanghai, China; ^3^ Shanghai Key Laboratory of Radiation Oncology, Shanghai, China

**Keywords:** radiomic, *KRAS*, prediction, local advanced rectal cancer, magnetic resonance imaging

## Abstract

**Background and Purpose:**

Locally advanced rectal cancer (LARC) is a heterogeneous disease with little information about *KRAS* status and image features. The purpose of this study was to analyze the association between T2 magnetic resonance imaging (MRI) radiomics features and *KRAS* status in LARC patients.

**Material and Methods:**

Eighty-three patients with *KRAS* status information and T2 MRI images between 2012.05 and 2019.09 were included. Least absolute shrinkage and selection operator (LASSO) regression was performed to assess the associations between features and gene status. The patients were divided 7:3 into training and validation sets. The C-index and the average area under the receiver operator characteristic curve (AUC) were used for performance evaluation.

**Results:**

The clinical characteristics of 83 patients in the *KRAS* mutant and wild-type cohorts were balanced. Forty-two (50.6%) patients had *KRAS* mutations, and 41 (49.4%) patients had wild-type *KRAS*. A total of 253 radiomics features were extracted from the T2-MRI images of LARC patients. One radiomic feature named X.LL_scaled_std, a standard deviation value of scaled wavelet-transformed low-pass channel filter, was selected from 253 features (*P*=0.019). The radiomics-based C-index values were 0.801 (95% CI: 0.772-0.830) and 0.703 (95% CI: 0.620-0.786) in the training and validation sets, respectively.

**Conclusion:**

Radiomics features could differentiate *KRAS* status in LARC patients based on T2-MRI images. Further validation in a larger dataset is necessary in the future.

## Introduction

Colorectal cancer (CRC) is one of the most prevalent cancers worldwide, and locally advanced rectal cancer (LARC) shows strong heterogeneity in real-world medical practice. The best treatment strategy for LARC patients still depends on the findings of further clinical trials.

*KRAS* mutation status has a strong relationship with the prognosis of CRC patients. In rectal cancer patients, *KRAS* mutant (*KRAS*-mut) patients have a worse prognosis (1), which emphasizes the importance of detecting *KRAS* status for prognostic evaluation and treatment strategy selection. Among metastatic CRC patients, *RAS* mutation is a negative predictive biomarker for treatment with epidermal growth factor receptor (*EGFR*) antibody therapies such as cetuximab and panitumumab (2). The role of *KRAS* status in stage III CRC patients is still being investigated. Years ago, researchers held the position that *KRAS* status was not associated with worse overall survival (OS) or disease-free survival (DFS) (3). With follow-up data maturing and treatments evolving, more studies are challenging this opinion based on the findings that *KRAS*-mut patients have worse OS and DFS (4, 5). Notably, most of these studies were conducted in CRC patients, and the number of patients with *KRAS* mutations was limited because their main research objective was immune-related biomarkers. As a result, the effect of targeted therapy in LARC patients remains unclear. From limited clinical trials, *KRAS* status was shown to be a significant predictor in multivariate analysis, and *KRAS*-mut patients had a worse response to neoadjuvant radiochemotherapy with worse OS than *KRAS* wild-type (*KRAS-*wild) patients (1, 6–8). Hence, information on *KRAS* mutation status has great meaning for physicians in predicting patient response to neoadjuvant chemotherapy and prognosis in practical medical treatment.

Because physicians will choose a targeted treatment strategy for metastatic CRC patients depending on *KRAS* status, efforts to obtain *KRAS* status from radiological images have been ongoing for years. To avoid invasive operations, an increasing number of studies on *KRAS* status and radiological image characteristics have been reported. For decades, several kinds of studies have been conducted on computed tomography (CT) (9)-based, positron emission tomography-CT (PET-CT) (10–17)-based and magnetic resonance imaging (MRI) (18)-based texture features to assess the relationships between genetic mutations and CRC metastatic rectal cancer patients (19). However, the results remain unstable and conflicting, and it is still unfortunate that the effects various radiological technologies remain unknown. Moreover, LARC patients are quite different from metastatic CRC patients in terms of treatment strategies and biological characteristics, especially the *KRAS* status. Therefore, specific studies on LARC patients deserve more attention.

Radiomics is a rapidly developing image acquisition and analysis technology that is used in various kinds of medical evaluations, especially in the diagnosis and prognosis of patients as well as the classification of different genotypes (20–22). As the first study focused on LARC patients, this study aimed to investigate whether MRI radiomics can predict *KRAS* status in LARC patients.

## Material and Methods

### Patient Profiles

A retrospective study of 83 LARC patients was performed. All patients had undergone an MRI examination of the primary tumor and *RAS* mutation analysis from our center. The inclusion criteria were as follows: (1) the primary tumor was proven to be rectal adenocarcinoma by biopsy; (2) MRI images could be acquired from our image database; and (3) clinical and treatment information could be acquired from our database. This study was approved by the Institutional Review Board of Fudan University Shanghai Cancer Center.

### MRI Image Acquisition

The primary tumor was imaged in a 3.0 Tesla (T) MRI (Signa Horizon, GE Medical Systems, Milwaukee, WI) using a phased-array body coil. The standard imaging protocol consisted of a sagittal T2-weighted (T2W) fast spin-echo image and an oblique axial thin-section T2W image, which was used for contouring the primary tumor.

### RAS Mutation Information

In RAS mutation analysis, tumor tissue was extracted from patients’ primary tumor sites by rectal biopsy or surgical resection, with formalin-fixed paraffin-embedded (FFPE) primary tumor sections produced using the QIAamp DNA FFPE Tissue Kit (Qiagen, Dusseldorf, Germany.). Mutations in *KRAS* (exons 2, 3, and 4), NRAS (exons 2, 3, and 4), and BRAF (V600E) were analyzed by the amplification refractory mutation system (AmoyDx Co., Xiamen, China) of samples from pathologic examination.

### Radiomic Feature Extraction

Regions of interest (ROIs) were distinguished from axial thin-section T2WI images and segmented by two experienced radiation oncologists (4 and 7 years of experience) in MIM software. The gross tumor was included in image delineation, and the air inside the rectum was carefully excluded.

The DICOM images and structure were sent to MATLAB (Math Works Inc.) for radiomics feature calculation and analysis. A total of 253 features were extracted from the ROI images. The features included grey features, texture features, shape features, fractal dimension features, and wavelet features. The detailed algorithm of these features was described by an updated quantitative radiomics standard from Alex (23).

### Feature Selection and Model Building

Clinical and radiomics features were extracted from the clinical database and DICOM images of the patients. For clinical features, the chi-square test was performed to compare the differences between two cohorts based on *KRAS* status. For features from T2WI images, the least absolute shrinkage and selection operator (LASSO) regression algorithm was performed for predictive feature selection and model establishment. The LASSO algorithm is a widely used method for the dimensionality reduction of high-dimensional data in artificial intelligence research and radiomics studies. Selected radiomics features were calculated for the radiomics score (rad-score) based on linear regression in the training cohort, and the formula was used in the validation cohort for rad-score calculation.

### Statistical Analysis

The distribution of continuous numeric data was affirmed by the Shapiro-Wilk test. The comparison of continuous numeric data was ascertained by the Kolmogorov-Smirnov test, and categorical data were compared by the chi-square test. The area under the curve (AUC) was used to depict the predictive accuracy of the model. The training set and validation set were divided according to a 7:3 ratio, and the concordance index (C-index) was presented for the result. The C-index can calculate the concordance of the model prediction and actual condition, whose value equals the AUC of the receiver operator characteristic (ROC) curve. And the decision curve analysis (DCA) was also applied. The best cut-off value was based on Youden’s index. A p-value <0.05 (z-value of 1.96) was considered statistically significant.

The packages involved in our research were listed as follow: tableone, MASS for table on creation, caret, lattice, dplyr, glmnet for data analysis and model building, ggplot2, pROC and rmda were used for result visualization and DCA analysis.

## Result

### Patient Characteristics

**Figure 1:**
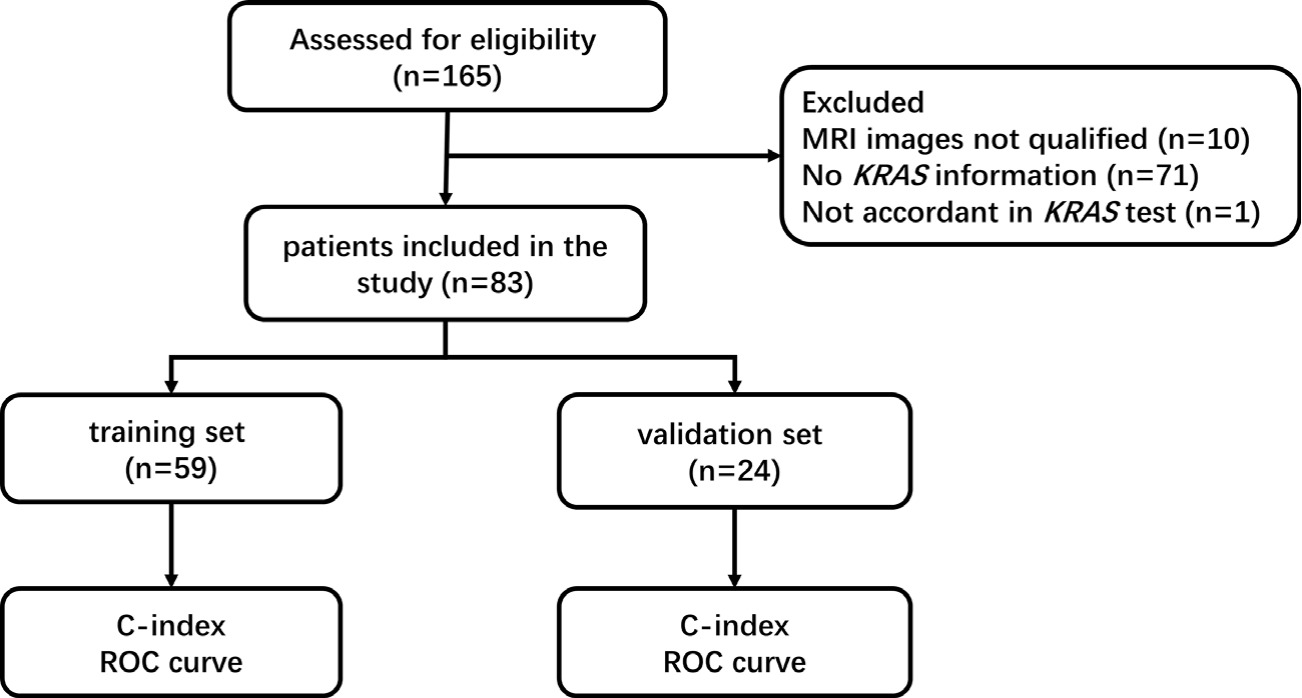
Flow chart of the study.

The summary profile of this research was shown in **Figure 1**. A total of 83 LARC patients were included in this study. Fifty-one (61.4%) of these patients were male, and the median age was 55 years, with a range of 29 to 87 years. Among all the patients, 74 (89.2%) were in stage III, and 7 (8.4%) patients were managed with a watch and wait (W&W) strategy. Seventy-six (91.6%) patients received neoadjuvant chemoradiation therapy, and 71 (87.7%) patients underwent surgery. For mutation status, 41 (49.4%) patients had mutations in the *KRAS* gene, and 2 (97.6%) patients had mutations in the *NRAS* and *BRAF* genes. The detailed characteristics are displayed in **Table 1**.

**Table 1:** Demographic and clinical characteristics of the *KRAS-*mut and *KRAS-*wild populations.

	Overall	*KRAS*-wild	*KRAS-*mut	*P-*value
Number	83	42	41	
Sex (%)				0.445
female	32 (38.6)	14 (33.3)	18 (43.9)	
male	51 (61.4)	28 (66.7)	23 (56.1)	
Age (mean (SD))				1
	55.95 (10.90)	55.95 (10.06)	55.95 (11.83)	
Distance to anus				0.477
	4.57 (1.96)	4.41 (2.04)	4.00[3.00-5.00]	
cT stage (%)				0.517
cT1	1 (1.2)	0 (0.0)	1 (2.4)	
cT2	4 (4.8)	3 (7.1)	1 (2.4)	
cT3	60 (72.3)	31 (73.8)	29 (70.7)	
cT4	18 (21.7)	8 (19.0)	10 (24.4)	
cN stage (%)				0.31
cN0	9 (10.8)	3 (7.1)	6 (14.6)	
cN1	23 (27.7)	10 (23.8)	13 (31.7)	
cN2	51 (61.4)	29 (69.0)	22 (53.7)	
C stage (%)				0.111
I	5 (6.0)	3 (7.1)	2 (4.9)	
II	4 (4.8)	0 (0.0)	4 (9.8)	
III	74 (89.2)	39 (92.9)	35 (85.4)	
MRF (%)				0.723
negative	34 (41.0)	18 (42.9)	16 (39.0)	
positive	35 (42.2)	16 (38.1)	19 (46.3)	
unknown	14 (16.9)	8 (19.0)	6 (14.6)	
EMVI (%)				0.611
negative	32 (38.6)	18 (42.9)	14 (34.1)	
positive	38 (45.8)	17 (40.5)	21 (51.2)	
unknown	13 (15.7)	7 (16.7)	6 (14.6)	
*KRAS* (%)				<0.001
wild type	42 (50.6)	42 (100.0)	0 (0.0)	
mutant	41 (49.4)	0 (0.0)	41 (100.0)	
*NRAS* (%)				0.485
wild type	81 (97.6)	40 (95.2)	41 (100.0)	
mutant	2 (2.4)	2 (4.8)	0 (0.0)	
*BRAF* (%)				0.485
wild type	81 (97.6)	40 (95.2)	41 (100.0)	
mutant	2 (2.4)	2 (4.8)	0 (0.0)	

The patients were divided into two categories based on *KRAS* status. For the overall clinical features, no obvious baseline differences were observed between the two cohorts (the details are displayed in **Tables 1** and **2**).

**Table 2:** Patient treatments and pathological characteristics.

	Overall	*KRAS-wild*	*KRAS-*mut	*P-*value
Watch and wait (W&W) (%)				0.019
non-W&W	76 (91.6)	35 (83.3)	41 (100.0)	
W&W	7 (8.4)	7 (16.7)	0 (0.0)	
Neoadjuvant chemoradiation therapy (NCRT) (%)				0.41
non-NCRT	7 (8.4)	2 (4.8)	5 (12.2)	
NCRT	76 (91.6)	40 (95.2)	36 (87.8)	
Surgery type (%)				0.024
APR	27 (32.5)	10 (23.8)	17 (41.5)	
palliative colon stoma	1 (1.2)	0 (0.0)	1 (2.4)	
Hartmann	7 (8.4)	5 (11.9)	2 (4.9)	
LAR	35 (42.2)	15 (35.7)	20 (48.8)	
trans-anal surgery	1 (1.2)	1 (2.4)	0 (0.0)	
W&W	7 (8.4)	7 (16.7)	0 (0.0)	
no surgery	5 (6.0)	4 (9.5)	1 (2.4)	
Tumor type (%)				0.485
adenocarcinoma	81 (97.6)	40 (95.2)	41 (100.0)	
mucinous adenocarcinoma	2 (2.4)	2 (4.8)	0 (0.0)	
Differentiation (%)				0.015
moderate	40 (48.2)	21 (50.0)	19 (46.3)	
poor	15 (18.1)	4 (9.5)	11 (26.8)	
unknown	21 (25.3)	10 (23.8)	11 (26.8)	
W&W	7 (8.4)	7 (16.7)	0 (0.0)	
ypT stage (%)				0.03
ypT0	7 (8.4)	1 (2.4)	6 (14.6)	
ypT1	1 (1.2)	0 (0.0)	1 (2.4)	
ypT2	12 (14.5)	7 (16.7)	5 (12.2)	
ypT3	47 (56.6)	21 (50.0)	26 (63.4)	
ypT4	1 (1.2)	1 (2.4)	0 (0.0)	
unknown	8 (9.6)	5 (11.9)	3 (7.3)	
W&W	7 (8.4)	7 (16.7)	0 (0.0)	
ypN stage (%)				0.025
ypN0	33 (39.8)	12 (28.6)	21 (51.2)	
ypN1	26 (31.3)	12 (28.6)	14 (34.1)	
ypN2	8 (9.6)	5 (11.9)	3 (7.3)	
unknown	9 (10.8)	6 (14.3)	3 (7.3)	
W&W	7 (8.4)	7 (16.7)	0 (0.0)	
ypTNM stage (%)				0.018
yp0	7 (8.4)	1 (2.4)	6 (14.6)	
ypI	4 (4.8)	1 (2.4)	3 (7.3)	
ypII	21 (25.3)	9 (21.4)	12 (29.3)	
ypIII	34 (41.0)	17 (40.5)	17 (41.5)	
unknown	10 (12.0)	7 (16.7)	3 (7.3)	
W&W	7 (8.4)	7 (16.7)	0 (0.0)	

### MR Radiomic Analysis

**Figure 2:**
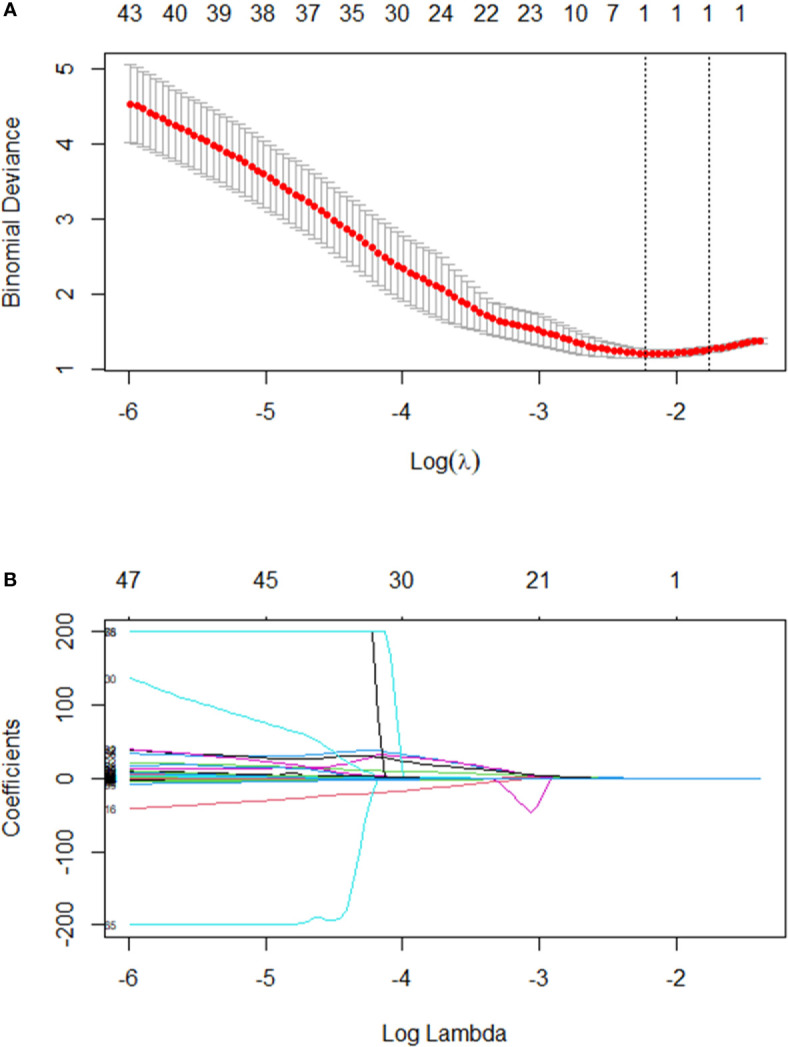
**(A)** Text features were selected by the LASSO regression model. The performance of the radiomics signature was assessed by the ROC curve and C-index. Tuning parameter (λ) selection used ten-fold cross-validation *via* the minimum criteria. The optimal value was calculated by the minimum criteria and the 1-standard error of the minimum criteria (the 1-SE criteria). A λ of 0.1782 with log(λ) - 1.75562 was chosen. **(B)** A LASSO coefficient profile plot was produced against the log(λ) sequence. In addition, one radiomics feature was selected.

After regression, one radiomic predictor was selected from 253 texture features. This feature is listed in **Table 3**. **Figure 2** presents the tuning parameter (λ) and the coefficient of LASSO regression. **Figure 2** presents the distribution of the selected parameter, X.LL_scaled_std, which is the standard deviation value of the scaled wavelet-transformed low-pass channel filter.

**Table 3:** Radiomics feature.

Feature	Coefficient
Intercept	-1.81132414
X.LL_scaled_std	0.04361241

### Characteristics of the Patients in the Training and Validation Sets

Based on the random selection of *KRAS-*mut and *KRAS*-wild patients, 59 (70%) patients were distributed to the training set, and 24 (30%) patients were distributed to the validation set. In the training set, there was no significant difference in the baseline information obtained based on the *KRAS* status cohort, but some differences appeared after neoadjuvant chemoradiation therapy according to the curative effect, as the ypTNM stage. In the validation set, no obvious differences were observed between the two cohorts. Detailed information is shown in **Tables 1**, **2** and **4**.

**Table 4:** Characteristics of patients in the training set and validation set.

	training set (n=59)			validation set (n=24)		
	*KRAS-wild*	*KRAS-*mut	*P-*value	*KRAS-wild*	*KRAS-*mut	*P-*value
Number	26	33		16	8	
Sex (%)			0.784			0.874
female	10 (38.5)	15 (45.5)		4 (25.0)	3 (37.5)	
male	16 (61.5)	18 (54.5)		12 (75.0)	5 (62.5)	
Age (mean (SD))	56.08 (10.19)	56.24 (11.61)	0.954	55.75 (10.16)	54.75 (13.47)	0.84
Distance to anus	4.40 (2.25)	4.60 (1.96)	0.726	4.43 (1.65)	4.50 [4.00,6.25]	0.313
cT stage (%)			0.801			0.105
cT 1	0 (0.0)	1 (3.0)		0 (0.0)	0 (0.0)	
cT 2	1 (3.8)	1 (3.0)		2 (12.5)	0 (0.0)	
cT 3	19 (73.1)	25 (75.8)		12 (75.0)	4 (50.0)	
cT 4	6 (23.1)	6 (18.2)		2 (12.5)	4 (50.0)	
cN stage (%)			0.104			0.57
cN0	1 (3.8)	6 (18.2)		2 (12.5)	0 (0.0)	
cN1	6 (23.1)	11 (33.3)		4 (25.0)	2 (25.0)	
cN2	19 (73.1)	16 (48.5)		10 (62.5)	6 (75.0)	
c stage (%)			0.163			0.794
I	1 (3.8)	2 (6.1)		2 (12.5)	0 (0.0)	
II	0 (0.0)	4 (12.1)		0 (0.0)	0 (0.0)	
III	25 (96.2)	27 (81.8)		14 (87.5)	8 (100.0)	
cMRF (%)			0.803			0.655
negative	14 (53.8)	15 (45.5)		4 (25.0)	1 (12.5)	
positive	9 (34.6)	14 (42.4)		7 (43.8)	5 (62.5)	
unknown	3 (11.5)	4 (12.1)		5 (31.2)	2 (25.0)	
cEMVI (%)			0.515			0.758
negative	14 (53.8)	13 (39.4)		4 (25.0)	1 (12.5)	
positive	9 (34.6)	16 (48.5)		8 (50.0)	5 (62.5)	
unknown	3 (11.5)	4 (12.1)		4 (25.0)	2 (25.0)	
ypTNM (%)			0.021			0.69
yp0	0 (0.0)	6 (18.2)		1 (6.2)	0 (0.0)	
ypI	1 (3.8)	3 (9.1)		0 (0.0)	0 (0.0)	
ypII	6 (23.1)	9 (27.3)		3 (18.8)	3 (37.5)	
ypIII	11 (42.3)	14 (42.4)		6 (37.5)	3 (37.5)	
unknown	3 (11.5)	1 (3.0)		4 (25.0)	2 (25.0)	
W&W	5 (19.2)	0 (0.0)		2 (12.5)	0 (0.0)	
*KRAS* (%)			<0.001			<0.001
wild type	26 (100.0)	0 (0.0)		16 (100.0)	0 (0.0)	
mutant	0 (0.0)	33 (100.0)		0 (0.0)	8 (100.0)	
*NRAS* (%)			0.904			1
wild type	25 (96.2)	33 (100.0)		15 (93.8)	8 (100.0)	
mutant	1 (3.8)	0 (0.0)		1 (6.2)	0 (0.0)	
*BRAF* (%)			0.904			1
wild type	25 (96.2)	33 (100.0)		15 (93.8)	8 (100.0)	
mutant	1 (3.8)	0 (0.0)		1 (6.2)	0 (0.0)	

### Model Efficacy in the Training Set and Validation Set

**Figure 3:**
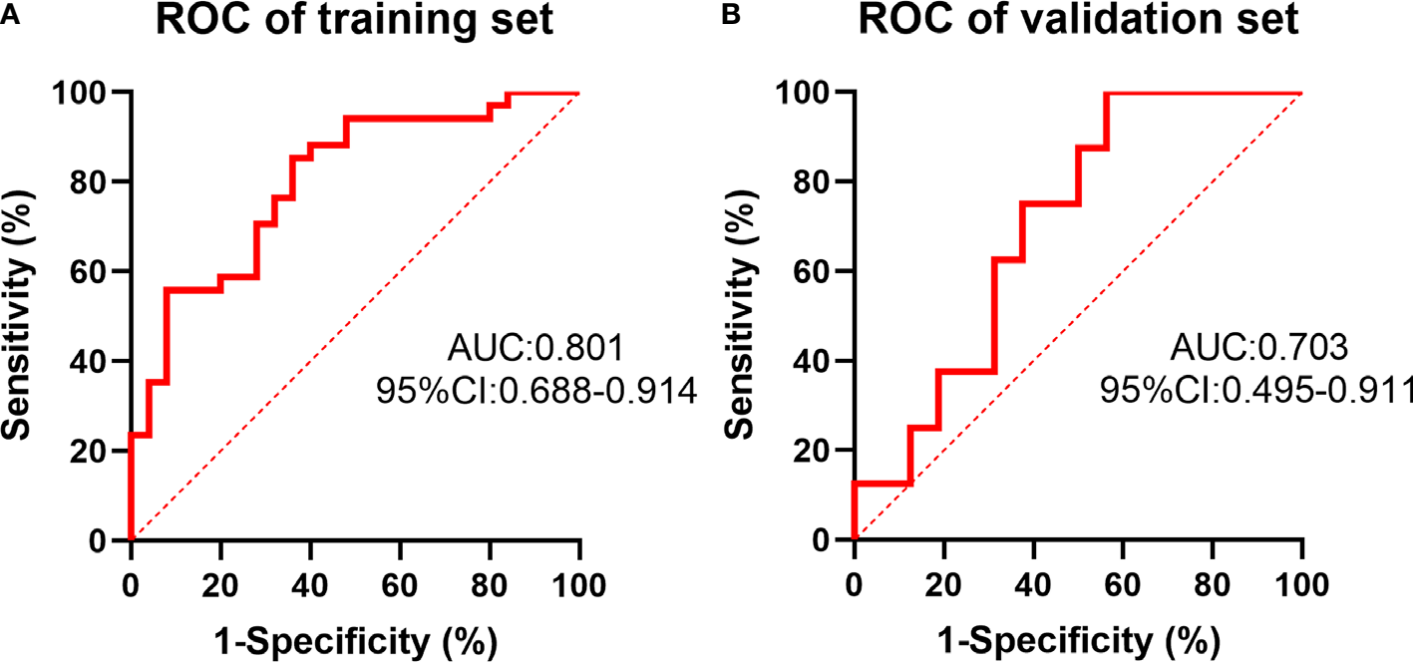
The receiver operating characteristic (ROC) curve of the prediction of *KRAS* status by the radiomics model in the training set **(A)** and validation set **(B)**.

In the training set, the predictive model achieved a C-index of 0.801 (95% confidence interval (CI) 0.772-0.830) based on 59 patients’ radiomic image data. The sensitivity and specificity for differentiating tumors with mutant *KRAS* status from those with wild-type status were 64% and 85.3%, respectively, based on the cut-off value of 0.452. In the validation set, this model achieved a C-index of 0.703 (95% CI 0.620-0.786), which was shown in **Figure 3**. The sensitivity and specificity for differentiation were 43.8% and 100%, respectively, based on the cut-off value of 0.365. The detailed information was listed in **Table 5**. The predictive effect of the radiomics model showed a stable performance in both the training set and validation set of LARC patients.

**Table 5:** Information of prediction performance.

	Training set (%)	Validation set (%)
Sensitivity	64.0	56.3
Specificity	85.3	100.0
Accuracy	76.3	62.5
Positive Predictive Value	76.2	52.9
Negative Predictive Value	76.3	100.0
C-index	80.1	70.3

**Figure 4:**
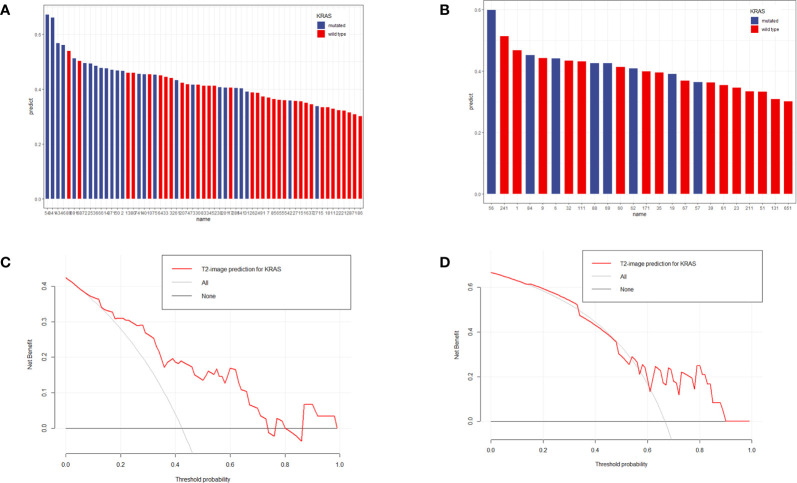
Distribution of prediction values in *KRAS-*mut and *KRAS-*wild patients in the training set **(A)** and validation set **(B)**. The y-axis measures the calculation value of the radiomic model. The blue columns represent actual *KRAS-*mut patients, and the red columns represent actual *KRAS-wild* patients. A higher column represents a higher value calculated by the model. According to the image, *KRAS-*mut patients more frequently obtained higher values than *KRAS-*wild patients. **(C**, **D)** represented the DCA analysis for the training set and validation set.

The specific values from the predictive model are listed in **Supplementary 1**. The distributions of patient *KRAS* status and predictive values are shown in **Figure 4**, which shows that patients with high prediction values had *KRAS*-mut status based on our prediction.

## Discussion

With years of development of targeted therapy, the targeted therapy strategy based on *KRAS* status has changed substantially. According to the treatment recommendation of the European Society for Medical Oncology (24), *KRAS* status is a negative predictive marker for anti-EGFR treatment selection. For LARC patients, even the anti-EGFR strategy did not have improved effects on *KRAS* wild-type patients in some clinical trials (6, 25); *KRAS* status still plays a role as a treatment effect biomarker, and LARC patients with the mutation have worse progression-free survival (PFS) (26). Based on the accumulation of evidence on LARC treatments in patients with different *KRAS* statuses, some clinical trials still present a promising curative effect. A pathological complete response (pCR) rate of 60% was achieved from neoadjuvant radiotherapy combined with capecitabine and sorafenib in *KRAS*-mut patients in phase II clinical trial (27). This finding hints that the determination of *KRAS* status is still important in LARC patients.

Nevertheless, the crucial role of *KRAS* has been reported for years, and the result of gene status can be revealed by only biopsy samples from colonoscopy or surgery in medical practice. Our research aims to detect *KRAS* status by radiomic to provide earlier information on gene expression as a noninvasive medical practice for patients.

To explore the value of radiomic features, we choose the T2-MRI images for radiomic features selection. As the treatments involving, MRI images have become the necessary tool for cancer staging. Because MRI images have the excellent ability for lymph node recognition, for neoadjuvant treatment selection, LARC patients are recommended to receive MRI examination at first diagnosis (28). Except for the great accessibility of MRI images, compared to other radiological tools, MRI images can also provide distinct tissue contrast for biological information and tumor border delineation.

We have found the value of X.LL_scaled_std, which can differentiate *KRAS* status with the best performance. This value was calculated to describe the standard deviation of the scaled wavelet-transformed low-pass channel filter. From the result, the higher value was observed in the *KRAS* mutant cohort. This deviation, as a value that can not detect visually, performed the heterogeneity of the ROI images. Previous research also revealed that higher heterogeneity can be observed in *KRAS* mutant tumor images, and they also found some value implied the shape characteristic of the tumor, not in our research (29). We believe that the morphological heterogeneity correlated to image reader strongly and tumor stage closely, which needs more researches to determine the delineation standard of ROI, and the role of shape will be clear.

Based on the value we found, the effect of our model is also comparable to other studies based on T2-images in rectal cancer. The prediction based on our research yielded a C-index of 0.703 (95% CI 0.620-0.786), Cui and his colleague got the AUC of 0.682 (95% CI 0.569–0.794) with 0.714 (95% CI 0.602–0.827) in their validation sets (29), and 0.886 from one dataset of oh and his colleagues (30). The researches based on T2-MRI images got a similar ability in the prediction of *KRAS* status, and some other studies have also focused on the same topic.

From the view of PET-CT, Pierre et al. assessed PET-CT for standardized uptake value (SUV), maximum SUV (SUVmax), mean SUV, skewness, SUV standard deviation, and SUV coefficient of variation (SUVcov). Both SUVcov and SUVmax showed an AUC of 0.65 (17). PET-CT is a great instrument for metabolic demonstration, and some studies presented a relationship between glucose metabolism and *RAS* status (31). In Pierre’s research, SUVmax was the most distinct parameter for *KRAS* status; in patients with *KRAS* mutations, SUVmax presented a higher latitude of elevation. However, these data did not reveal the same correlation between SUVmax and *KRAS* status (12, 13). SUVcov was also a latent parameter for *KRAS* recognition in the PET-CT results. Even though the predictive efficacy of treatment based on SUVcov baseline has been shown for neoadjuvant rectal cancer treatment (32), the whole PET-CT parameters show a low sensitivity and specificity of 0.66 (95% CI 0.60–0.73) and 0.67 (95% CI 0.62–0.72) (14), respectively. In summary, PET-CT is a direct demonstration of tumor metabolism but still cannot uncover the strong relationship between the parameters of SUV and *KRAS* status based on the current evidence.

In addition to studies on PET-CT, some researchers have also focused on CT images and gene characteristics. Lei Yang (9) tried to use CT-based radiomics signatures to predict gene mutations. In their study, five feature sets were extracted from the primary set that was established for model building. The five feature sets included the shape set, grey-level histogram feature set, grey-level co-occurrence matrix feature set, grey-level run-length matrix feature set, and overall feature set. For the validation of the CT-based model, the accuracy of the validation cohort was 0.750 (95% CI, 0.623-0.845), with a sensitivity of 0.686 and a specificity of 0.857. The value of radiomics was highly related to genetic mutations, with P<0.001 and odds ratio (OR) 11.18 (95% CI, 2.88-43.46) in the validation cohort.

Most of these studies focused on CRC patients, and some studies focused on rectal cancer for further research. Yang tried to differentiate *KRAS* status by CT-based radiomics signatures, and the AUC was 0.829 in the validation set (9). Xu summarized the *KRAS*-related features in rectal cancer. The mean values of six texture parameters were significantly higher in the *KRAS*-mut group than in the *KRAS*-wild group. The AUC values of the texture features ranged from 0.703 to 0.813 and used T2-MRI radiomics to predict *KRAS* status, and they had an accuracy of 81.7% for the decision tree (18). However, the sample size of their research was 60, and 12% of patients were stage IV (M1), so it is limited in sample size and cohort consistency.

LARC patients have specific clinical characteristics, and T2-MRI radiomics features deserve more exploration based on the limited study focus on such technology.

Our study also has some limitations. First, external validation needs to be performed in the future to consolidate the results. Second, in addition to radiomics, deep learning and other artificial intelligence technologies could be used in image data analysis and model establishment, which may further improve the results. Third, more MRI images with latent bio-information, for example, enhanced sequence and DWI can be achieved for further exploration with KRAS status, which may increase the predictive precision.

To summarize, our study focused on the exploration of the relationship between T2-MRI and *KRAS* status in LARC patients. We present the strong value of radiomics in the prediction of *KRAS* status before neoadjuvant chemoradiation therapy and provide a non-invasive method for further targeted therapy strategy selection.

## Data Availability Statement

The original contributions presented in the study are included in the article/**Supplementary Material**. Further inquiries can be directed to the corresponding author.

## Ethics Statement

The studies involving human participants were reviewed and approved by the ethics committee of the Shanghai Cancer Center.

## Author Contributions

ZZ and LS contributed to conception and design. YW and ZZ collected the data. ZZ analyze and interpreted of data. All authors listed have made a substantial, direct, and intellectual contribution to the work and approved it for publication.

## Funding

Shanghai Committee of Science and Technology Fund (19DZ1930902) and Xuhui District Artificial Intelligence Medical Hospital Cooperation Project (2020-009).

## Conflict of Interest

The authors declare that the research was conducted in the absence of any commercial or financial relationships that could be construed as a potential conflict of interest.

## Abbreviations

LARC, locally advanced rectal cancer; MRI, magnetic resonance imaging; CT, computed tomography; PET-CT, positron emission tomography-CT; LASSO, least absolute shrinkage and selection operator; AUC, area under the receiver operator characteristic curve; ROC, receiver operator characteristic; DCA, decision curve analysis; CRC, colorectal cancer; *KRAS*-mut, *KRAS* mutant; *KRAS-*wild, *KRAS* wild-type; OS, overall survival; DFS, disease-free survival; ROI, Region of interest.
