# Supplementary material for: MRI Radiomics Signature as a Potential Biomarker for Predicting KRAS Status in Locally Advanced Rectal Cancer Patients
Source: Front Oncol. 2021 May 7;11:614052. doi: 10.3389/fonc.2021.614052 (PMC8138318; doi:10.3389/fonc.2021.614052)
Supplement: Supplementary file 2 [file Table_2.docx]

Supplementary table2:

We list the *P*-values of the Shapiro-Wilk normality test for our cohorts involved in the research in supplementary table2.

And the distance to the anus in *KRAS* mutant patients followed a non-normal distribution, and we presented them in a quartile form.

Supplementary table2 *P-*values for normality test of every cohorts

|  | Overall | KRAS-wild | KRAS-mutant |
| --- | --- | --- | --- |
| Overall the cohort | | | |
| Age | 0.899 | 0.892 | 0.983 |
| Distance to anus | 0.075 | 0.323 | 0.029 |
| Training set | | | |
| Age | 0.280 | 0.696 | 0.517 |
| Distance to anus | 0.162 | 0.472 | 0.071 |
| Validation set | | | |
| Age | 0.203 | 0.621 | 0.507 |
| Distance to anus | 0.379 | 0.200 | 0.041 |
