# Supplementary material for: MRI Radiomics Signature as a Potential Biomarker for Predicting KRAS Status in Locally Advanced Rectal Cancer Patients
Source: Front Oncol. 2021 May 7;11:614052. doi: 10.3389/fonc.2021.614052 (PMC8138318; doi:10.3389/fonc.2021.614052)
Supplement: Supplementary file 3 [file DataSheet_1.docx]

Supplementary Fig1:

| A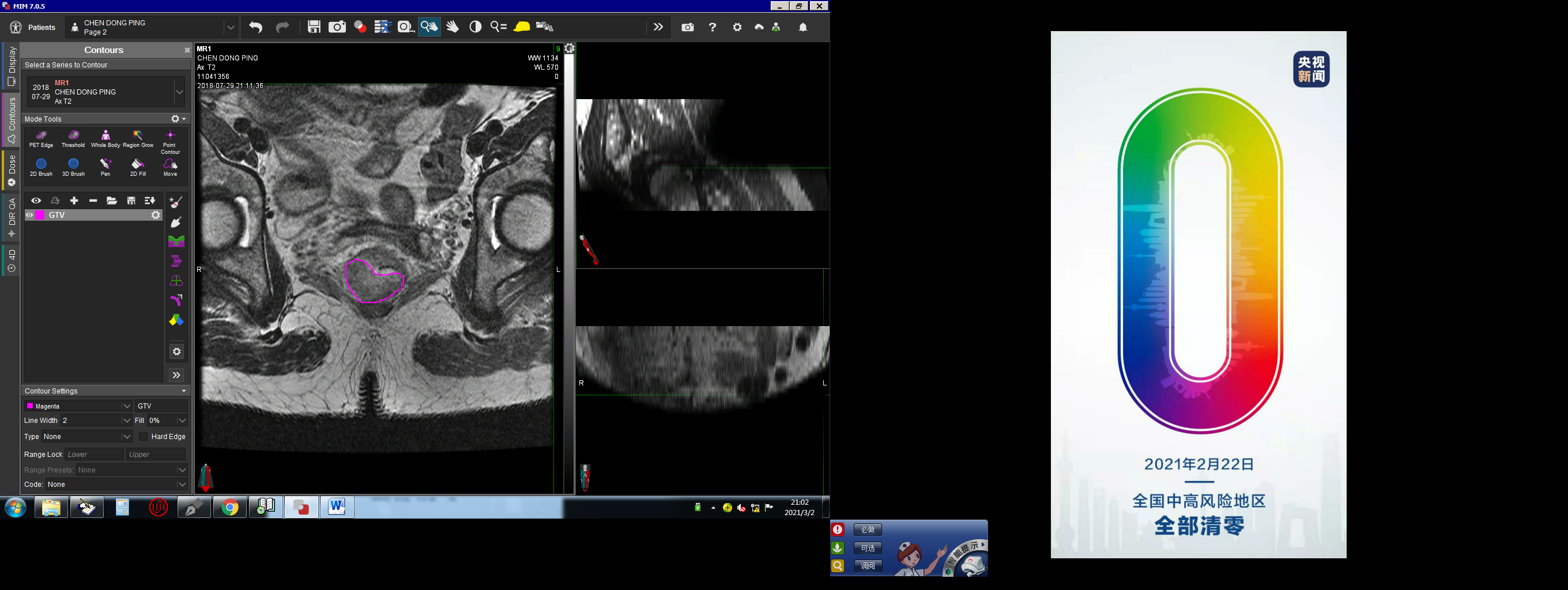 | B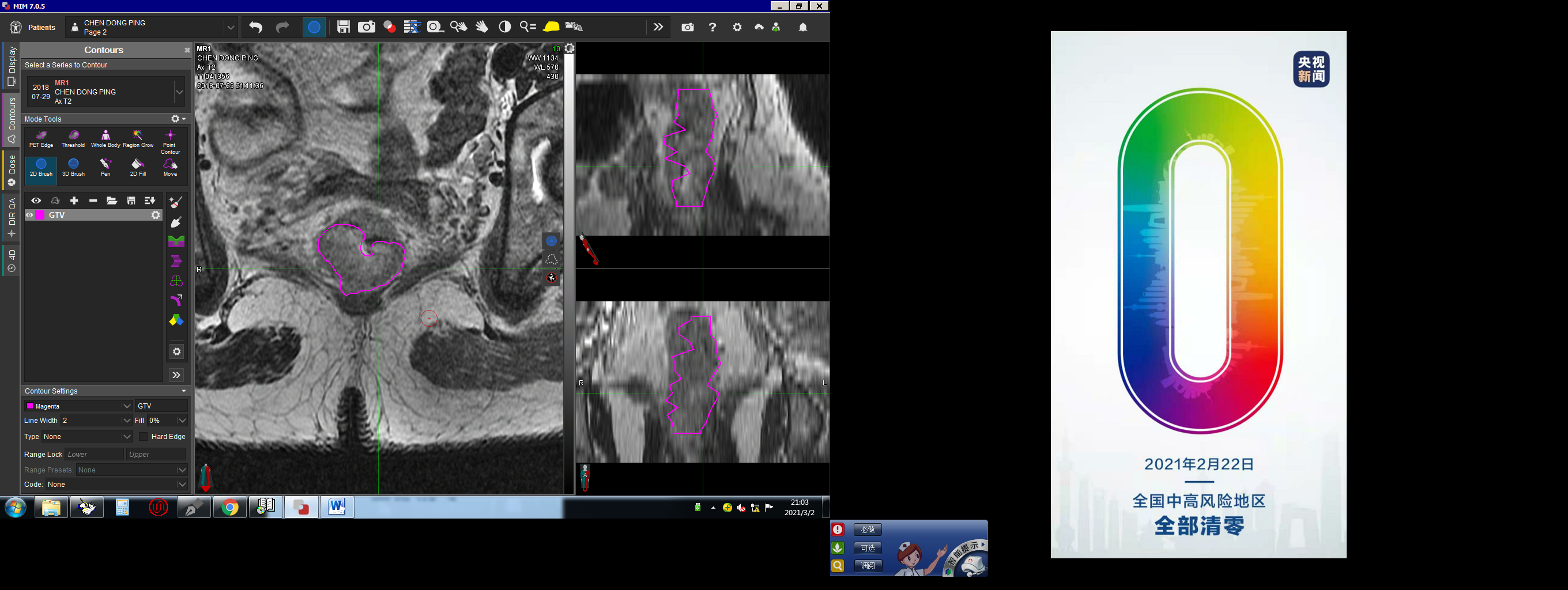 |
| --- | --- |
| Supplementary Fig1:Examples of ROI images. (A) An delineation of ROI. (B) An delineation of ROI in the following layer. | |
